# Plasma Apolipoprotein A-V Predicts Long-term Survival in Chronic Hepatitis B Patients with Acute-on-Chronic Liver Failure

**DOI:** 10.1038/srep45576

**Published:** 2017-03-30

**Authors:** En-Qiang Chen, Meng-Lan Wang, Dong-Mei Zhang, Ying Shi, Do-Bo Wu, Li-Bo Yan, Ling-Yao Du, Ling-Yun Zhou, Hong Tang

**Affiliations:** 1 Center of Infectious Diseases, West China Hospital, Sichuan University, Chengdu, Sichuan 610041, P. R. China; 2 Division of Infectious Diseases, State Key Laboratory of Biotherapy, Sichuan University, Chengdu, Sichuan 610041, P. R. China

## Abstract

Hepatitis B virus-related acute-on-chronic liver failure (HBV-ACLF) is a life-threatening condition, and the lipid metabolism disorder is common in the development of this disease. This prospective observational study aimed to define the characteristics of plasma apolipoprotein A-V (apoA-V) in long-term outcome prediction of HBV-ACLF, and a total of 330 HBV-ACLF patients were included and followed for more than 12 months. In this cohort, the 4-week, 12-week, 24-week and 48-week cumulative mortality of HBV-ACLF was 18.2%(60/330), 50.9%(168/330), 59.7%(197/330) and 63.3%(209/330), respectively. As compared to survivors, the non-survivors had significantly lower concentrations of plasma apoA-V on admission. Plasma apoA-V concentrations were positively correlated with prothrombin time activity (PTA), and negatively correlated with interleukin-10, tumor necrosis factor-α, and iMELD scores. Though plasma apoA-V, PTA, total bilirubin(TBil) and blood urea nitrogen(BUN) were all independent factors to predict one-year outcomes of HBV-ACLF, plasma apoA-V had the highest prediction accuracy. And its optimal cutoff value for one-year survival prediction was 480.00 ng/mL, which had a positive predictive value of 84.68% and a negative predictive value of 92.23%. In summary, plasma apoA-V decreases significantly in non-survivors of HBV-ACLF, and it may be regarded as a new predictive marker for the prognosis of patients with HBV-ACLF.

Acute-on-chronic liver failure (ACLF) is an acute and rapid deterioration of previously well-compensated chronic liver disease^1^,^2^. In China and most other Asian countries, hepatitis B virus (HBV)-associated ACLF (HBV-ACLF) patients account for more than 50% of ACLF patients, owing to a high prevalence of chronic HBV infection^3^. Though nucleos(t)ide analog therapy, bioartificial liver support systems and stem cell transplantation have been investigated in HBV-ACLF, the long-term survival of HBV-ACLF is still not improved significantly over the past few decades, because majority of them were diagnosed in the very late stages and missed the best treatment time^4^,^5^. Thus, this is a urgent need to establish sensitive and specific bio-markers for early warning and prediction of HBV-ACLF progression, and the latter is critical for timely identifying high-risk patients and making appropriate treatment strategies^6^,^7^,^8^.

Previous studies have shown that severe liver damage can induce a wide array of metabolic abnormalities, and lipid metabolism disorder is common in the early stages of liver failure^9^,^10^,^11^,^12^, which may be associated with the abnormal expression of the apolipoprotein (apo) genes^13^,^14^. As major component of plasma lipoproteins, apolipoproteins are mainly expressed in liver and secreted into plasma, and play important roles in physiology and pathogenicity of lipid *in vivo*^15^. Because they could act as the ligand for targeting lipoprotein receptors and activating a variety of lipoprotein metabolism enzymes, thus regulating the synthesis, secretion, maturation and degradation of lipoproteins. Studies also indicated that the hepatic lipoproteins expression are also influenced by the change of cell microenvironment, and their expression levels may reflect the degree of liver damage and inflammation. For example, plasma apoB and apoA-I levels are decreased significantly in critically ill patients with altered coagulation and inflammation^16^.

ApoA-V is an apolipoprotein that was independently discovered by Pennacchio *et al*. and van der Vliet *et al*. as a result of their investigations on new open-reading frames on chromosome 11q23 in the ApoA-I/ApoC-III/ApoA-IV gene cluster^17^,^18^. As the newest member of the Apo A class of proteins, apoA-V is expressed in liver and secreted into plasma. Since its discovery, ApoA-V has been shown to be a key regulator of plasma triglycerides (TG), although its plasma concentration is very low compared to other known apoproteins^19^. In our previous animal studies with fulminant hepatic failure, the changes of the plasma concentrations and mRNA expression of apoA-V were more significantly than that of other apoproteins^9^. Thus, we hypothesize that low levels of apoA-V are associated with a more pronounced lipid metabolism disorder and liver damage in patients with HBV-ALCF. In this prospective observational study, we aimed to define the characteristics of plasma apoA-V in HBV-ACLF and investigate whether the concentration of apoA-V was associated with prognosis of patients.

## Patients and Methods

### Study design and patients

This was a prospective study and it was registered with the Chinese Clinical Trial Registry (http://www.chictr.org) (Identifier: ChiCTR-PRCH-13002984). All HBV-ACLF patients were recruited from West China Hospital of Sichuan University between January 2013 to December 2014. And the diagnosis of HBV-ACLF was mainly based on the following criteria: (i) pre-existing CHB without evidence of cirrhosis on ultrasound imaging or CT scan; (ii) progressive hyperbilirubinaemia, defined as a >50% increase in bilirubin or up to a level of >280 μmol/L within 4 weeks; (iii) prothrombin activity (PTA) ≤ 40% or INR ≥ 1.5. In this study, HBV-ACLF patients were excluded if they were infected with other hepatitis viruses (including A, C, D and E) or human immunodeficiency virus (HIV); or had evidences of drug-induced liver injury, alcoholic liver disease, autoimmune liver diseases, diabetes, severe systemic illnesses, hepatocellular carcinoma and other tumors.

According to the design of this study, all HBV-ACLF patients would be followed up for 12 months to identify the status of long-term clinical outcomes. The death or survival information of patients were obtained from electronic medical records in hospital or telephone follow-up. If the survival information could not be obtained from above two ways, we would refer to the Chinese household registration management system in which the death is registered.

This study conformed to the ethical guidelines of the 1975 Declaration of Helsinki. Approval of this study was obtained from the Ethics Committee of West China Hospital of Sichuan University(IRBref#201303), and informed consent was obtained from each patient or his/her legal guardian.

### Laboratory examination

The blood parameter, serum biochemical and electrolyte indices were detected by blood analyzer and automatic biochemical analyzer using the standard procedures (Olympus AU5400, Olympus Corporation, Tokyo, Japan). HBV serological markers were evaluated by electrochemiluminescence immunoassay (Elecsys; Roche Diagnostics, China). Serum levels of HBV DNA were measured by the real-time polymerase chain reaction (PCR). The plasma IL-10, TNFα, and apoA-V levels were determined using the enzyme linked immunosorbent assay (ELISA) method according to the manufacturer’s protocols.

### Statistical Analyses

Quantitative variables were expressed as the mean ± standard deviation or the median with interquartile ranges (IQR), and categorical variables as absolute and relative frequencies. The t-test or the nonparametric Mann–Whitney U-test were performed to calculated differences between quantitative data; while chi-squared test or Fisher’s exact test were performed to calculated differences between qualitative data. Correlation between two quantitative variables was analyzed using spearman’s bivariate correlation, and the correlation is significant at the 0.01 level (2-tailed). Independent factors associated with the outcome of HBV-ACLF were assessed using Cox proportional hazard regression model, where significant variables in univariate analysis were included in this model. In this study, the Area Under Receiver Operating Characteristics (AUROC) was used to assess the accuracy of the independent factors and to identify optimal cutoff value for the prediction of survival in HBV-ACLF patients. The optimal cutoff values were chosen based on a maximum sum of sensitivity and specificity. A *P* value less than 0.05 was considered to indicate statistical significance. All statistical analyses were done with SPSS Version 18.0 (SPSS, Chicago, IL), and figures were drawn using GraphPad Prism 6 (GraphPad Software Inc., California, USA).

## Results

### Baseline Characteristics

The detailed baseline characteristics of the 330 HBV-ACLF patients were shown in Table 1. The mean age of patients was 43.38 years, with a remarkable male predominance (77.08%). The median value of serum TBil was 360.65 mmol/L, and mean PTA was 24.17 ± 8.29%. There were 190 patients with negative HBeAg(57.58%), and the media HBV DNA serum level was 4.44 log10 copies/mL. Only 10 patients (3.03%) had received antiviral treatment before admission, and high to 277 patients (83.92%) had received antiviral treatment after admission. And 151 patients (45.76%) received plasmapheresis after admission. Among those HBV-ACLF patients, 60 patients (18.2%) died within 4 weeks, 168 patients (50.9%) died within 12 weeks, 197 patients (59.7%) died within 24 weeks, and 209 patients (63.33%) died within 48 weeks.

### Serum apolipoproteins in HBV-ACLF

**Figure 1:**
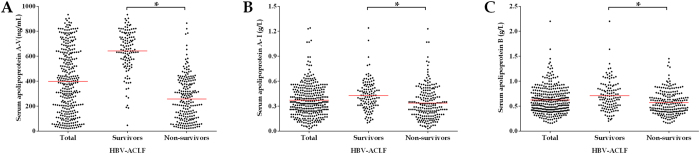
The distribution of serum apolipoproteins in HBV-ACLF patients. (**A**) apolipoprotein A-V; (**B**): apolipoprotein A-I; (**C**): apolipoprotein **B**.

In this study, the plasma levels of apoA-V, apoA-I and apoB were detected in all 330 HBV-ACLF patients, and the mean value was 398.58 ng/mL for plasma apoA-V, 0.37 g/L for apoA-I, and 0.62 g/L for apoB (Fig. 1). As compared to non-survivors at month 12, the serum levels of apoA-V was statistically significant higher in survivors (641.91 ± 168.20 ng/mL *vs.* 257.71 ± 175.48 ng/mL, *P* < 0.001). The similar findings were also observed in serum apoA-I (0.43 ± 0.18 g/L vs. 0.34 ± 0.20 g/L, *P* < 0.001) and apoB (0.71 ± 0.30 g/L vs. 0.57 ± 0.23 g/L, *P* < 0.001) between survivors and non-survivors.

### The correlation of serum apoA-V levels with clinical variables in HBV-ACLF

**Figure 2:**
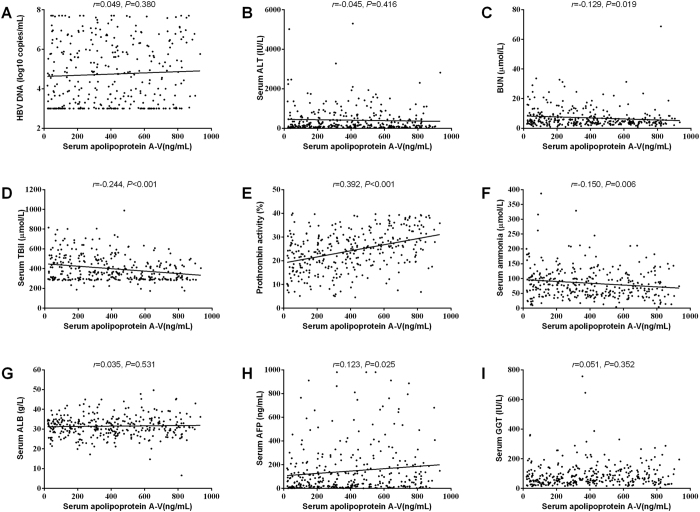
The correlation of serum apolipoprotein A-V levels with general laboratory variables. Correlation is significant at the 0.01 level (2-tailed).

**Figure 3:**
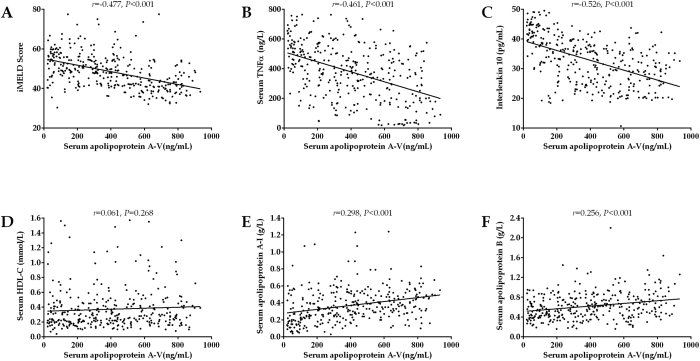
The correlation of serum apolipoprotein A-V levels with iMEDL score (**A**) and other laboratory variables (**B~F**). Correlation is significant at the 0.01 level (2-tailed).

In order to better understand the value of apoA-V in HBV-ACLF, we further analyzed the correlation of apoA-V with different clinical variables. As showed in Fig. 2 and Fig. 3, the serum levels of apoA-V were negatively correlated with BUN(*r* = −0.129, *P* = 0.019), TBil (*r* = −0.244, *P* < 0.001), ammonia (*r* = −0.150, *P* = 0.006), iMEDL score(*r* = −0.477, *P* < 0.001), TNFα(*r* = −0.461, *P* < 0.001) and IL10 (*r* = −0.526, *P* < 0.001); and postively correlated with PTA(*r* = 0.392, *P* < 0.001), AFP (*r* = 0.123, *P* = 0.025), apoA-I (*r* = 0.298, *P* < 0.001), and apoB (*r* = 0.256, *P* < 0.001). However, the intensity of correlations were relative weak for apoA-V with serum BUN, TBil, ammonia, AFP, apoA-I and apoB. Additionally, the serum levels of apoA-V were not significantly correlated with serum HBV DNA, ALT, ALB, GGT and HDL.

### Factors for predicting the survival of HBV-ACLF

The univariate analysis result of the survival was shown in Table 1. As compared to non-survivors, survivors seemed to be younger (*P* < 0.001) and had higher PTA (*P* < 0.001), lower TBil (*P* < 0.001), lower Amon (*P* = 0.003), lower BUN (*P* < 0.001), lower Cr (*P* < 0.001), higher AFP (*P* = 0.004), less bacterial infection (*P* = 0.003), higher IL10 (*P* < 0.001) and TNFα (*P* < 0.001), and lower iMELD socre (*P* < 0.001). However, there were no statistically significant differences in serum HBV DNA (*P* = 0.220), HBeAg state (*P* = 0.281), serum ALT(*P* = 0.061), ALB (*P* = 0.129), GGT (*P* = 0.521), TG (*P* = 0.051), and LDL (*P* = 0.069) levels between non-survivors and survivors. Additionally, the rates of antiviral therapy before (*P* = 0.180) and after (*P* = 0.656) admission, and the rate of plasmapheresis after admission (*P* = 0.754) were all similar between non-survivors and survivors.

In multivariate analysis (Table 2), plasma apoA-V (*P* < 0.001), PTA (*P* < 0.001), TBil (*P* < 0.001) and BUN (*P* < 0.001) were all significant independent factors for predicting the survival of HBV-ACLF. An unexpected observation in the multivariate analysis was that serum iMELD score (*P* = 0.055), AFP (*P* = 0.069), IL10 (*P* = 0.308), TNFα (*P* = 0.382) and Amon (*P* = 0.156) were not predictors of the outcomes of HBV-ACLF.

### ROC analysis of serum apoA-V in predicting the survival of HBV-ACLF

**Figure 4:**
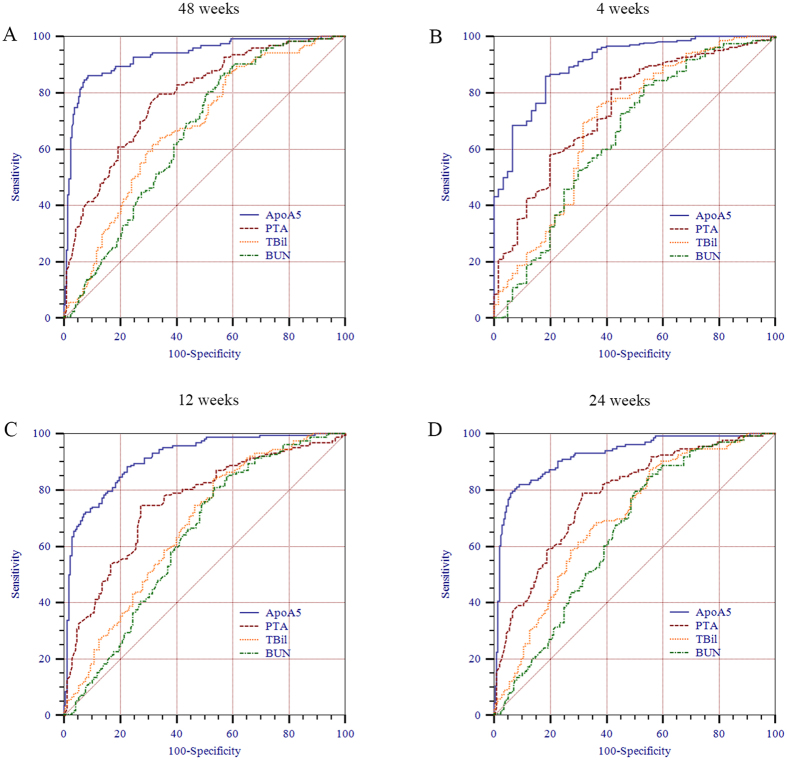
Accuracy of the serum apoA-V as compared to that of PTA, TBil and BUN in predicting the survival of the patients with HBV-ACLF at different time point using Receiver operating characteristic (ROC) curves. (**A**) for 48 weeks; (**B**) for 4 weeks; (**C**) for 12 weeks; (**D**) for 24 weeks.

The ROC curves analysis was performed to compare the AUROCs for serum apoA-V, PTA, TBil and BUN levels in predicting the outcomes of HBV-ACLF patients at 4, 12, 24 and 48 weeks. In predicting the 1-year survival of patients (Fig. 4A), the AUROC values were 0.929 (95% CI 0.896–0.954) for apoA-V, 0.792 (95%CI 0.745–0.835) for PTA, 0.674 (95%CI 0.620–0.724) for TBil, and 0.664 (95%CI 0.610–0.715) for BUN.

And similar findings were also observed in predicting the 4-week (Fig. 4B), 12-week (Fig. 4C) and 24-week (Fig. 4D) survival of patients. And these findings suggested serum apoA-V had the highest performance for the prediction of the survival of HBV-ACLF.

**Figure 5:**
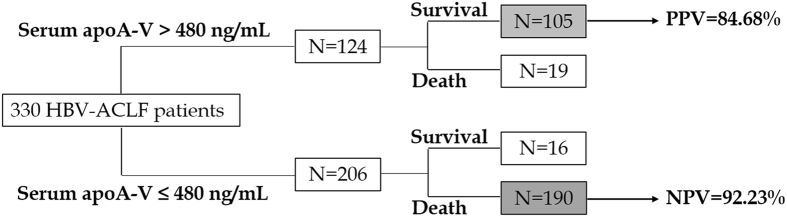
Predictive value of admission serum apoA-V level (cut-off value of >480 ng/mL) on the survival of HBV-ACLF.

In this study, the optimal cut-off values of serum apoA-V suggested by ROC curves analysis was 480.00 ng/mL. At the time of hospital admission, 124 HBV-ACLF patients had serum apoA-V >480 ng/mL. Among these patients, 105 patients survived. By contrast, 190 of the 206 HBV-ACLF patients who had serum apoA-V ≤ 480 ng/mL died in hospital or after being discharged from hospital. As shown in Fig. 5, the cut-off value of >480.00 ng/mL (85.95% sensitivity and 90.91% specificity) in serum apoA-V had a positive predictive value (PPV) of 84.68% and a negative predictive value (NPV) of 92.23%.

## Discussion

In this study, the prognosis of HBV-ACLF patients was poor, and only 36.67% patients survived for more than 12 months. As mentioned above, the lipid metabolism disorder is common in the development of HBV-ACLF, and our previous animal study showed that the changes of the plasma concentrations of apoA-V were more significantly than that of other apoproteins (data unshown). So in this prospective observational study, we further analyzed the levels of plasma apoA-V and its correlation with various clinical parameters in a real-life cohort of HBV-ACLF patients. And the major findings from present study are: (1) a significantly lower concentrations of plasma apoA-V in non-survivals as compared to survivals; (2) plasma concentrations of apoA-V on admission is a good predictor of HBV-ACLF outcomes; (3) the optimal cutoff value of plasma apoA-V was 480.00 ng/mL with a PPV of 84.68% and a NPV of 92.23%.

In past decade, artificial liver has been regarded as potential therapy for liver failure patients^20^. However, the prognosis of HBV-ACLF is still not good^21^. In this cohort, about 45.76% of patients had received plasmapheresis after admission, but still high to 63.33% patients died within 12 months after diagnosis. Though this rate seemed to be higher than that reported in similar studies, we didn’t think they were contradictory to each other. Because the outcomes of HBV-ACLF in majority of previous studies were calculated either at the time of hospital discharge or at 3 months after the disease onset, which determined a relatively low mortality rate. Thus, how to reduce the mortality of HBV-ACLF was still an urgent problem that needed to be solved. Though the poor prognosis of HBV-ACLF was due to many reasons, the failure to early warning and late diagnosis should be the key reason under the present medical conditions^22^.

As a novel member of the class of exchangeable apolipoprotein, a portion of hepatic-derived apoA-V is secreted into plasma and functions to facilitate lipoprotein lipase-mediated TG hydrolysis, another portion is recovered intracellularly, in association with cytosolic lipid droplets^19^,^23^. In previous studies, the loss of apoA-V was reported to be positively correlated with risk of cardiovascular disease^24^. In our previous animal experiment, we found a disorder of lipid metabolism in liver failure mouse models, and the expression of some apolipoproteins (such as apoA-V, apoA-I, apoB, etc.) were significantly declined. In present, we found that the plasma concentration of apoA-V was not only declined in patients with HBV-ACLF, but also associated with the outcomes of HBV-ALCF patients. The similar results were also observed in plasma apoA-I and apoB. These findings had confirmed that the disorder of apolipoprotein not only existed in cardiovascular diseases, but also was common in liver failure.

In this study, we firstly reported that plasma apoA-V concentrations were associated with the clinical outcomes of HBV-ACLF patients, and the risk of deaths increased significantly with the decline of plasma apoA-V concentrations. In past few years, many other blood variables (including PTA, plasma bilirubin, etc.) also were investigated and reported for predicting the outcomes of HBV-ACLF^6^,^25^; however, majority of them were difficult to take into account both survival and death predictions. On the contrary, plasma apoA-V has both good positive predictive value (84.68%) for survival and negative predictive value (92.23%) for death in this cohort, with an optimal cutoff value of plasma apoA-V at 480.00 ng/mL. So, apoA-V would be a promising new variable for prognosis prediction of HBV-ACLF in future.

It was worth to mention that when ACLF were diagnosed, patients and their families generally expected their physicians to provide them with an educated guess about the probability of survival, and some of families may choose to give up treatment because of uncertainty of clinical outcomes and huge medical costs. Now, to a certain degree, our findings may help physicians answer this question, and help patients and their families improve their confidence and compliance to the long-term comprehensive therapies, and the latter is helpful and important to improve the cure rate of ACLF in real-life clinical practice.

As we known, nutritional and metabolic disorders are common in CHB patients, which could aggravate liver injury by influencing the microenvironment of hepatic cells. When the damage to hepatic cells is out of control, some CHB patients would develop into liver failure. Lots of studies have shown that the disorder of lipid metabolism can trigger harmful oxidation/non-oxidative metabolic pathways and thus produce a large number of toxic metabolites accumulated in liver tissues^26^,^27^. Those toxic metabolites would easily induce endoplasmic reticulum stress and mitochondrial dysfunction which further promote the burst generation of inflammatory cell molecules, and finally induce and aggravate liver injury^27^,^28^. At present, the disorder of lipid metabolism has been regarded as one of the important factors that affect the progression of chronic liver diseases. In addition, more and more studies also indicated that the timely intervention of the intrahepatic lipid metabolic disorder may not only meet the needs of the body’s energy, but also promote the repair and regeneration of damaged liver cells, enhance the liver’s resistance to infections and toxins, and effectively reduce the occurrence of complications. So based on the findings of the above basic studies, we speculated that apoA-V not only could act as a predictive marker for the prognosis of HBV-ACLF, but also may be used as a target for clinical intervention. Of course, this is just a speculation now, more studies are needed to confirm it.

In summary, the prognosis of HBV-ACLF is still not satisfied at present. Plasma concentration of apoA-V decreases significantly in non-survivors of HBV-ACLF, and it may be regarded as a new predictive marker for the prognosis of HBV-ACLF. As an effective predictive marker of HBV-ACLF prognosis, a relative higher plasma apoA-V concentration help a considerable amount of patients and their families establish the confidence to fight with diseases, and the latter is extremely helpful and important to improve the cure rate of ACLF in real-life clinical practice.

## Additional Information

**How to cite this article**: Chen, E.-Q. *et al*. Plasma Apolipoprotein A-V Predicts Long-term Survival in Chronic Hepatitis B Patients with Acute-on-Chronic Liver Failure. *Sci. Rep.* **7**, 45576; doi: 10.1038/srep45576 (2017).

**Publisher's note:** Springer Nature remains neutral with regard to jurisdictional claims in published maps and institutional affiliations.

## Figures and Tables

**Table 1:** Baseline characteristics of survivors and non-survivors among HBV-ACLF patients.

	Total (n = 330)	Survivor (n = 121)	Death (n = 209)	*P*-Value
Age (years)	43.38 ± 11.72	40.31 ± 11.07	45.16 ± 11.74	<0.001
Gender (M/F)	290/40	104/17	186/23	0.414
HBeAg (+/−)	140/190	56/65	84/125	0.281
HBV DNA (log_10_copies/mL)	4.44 (3.11)	4.20 (2.80)	4.72 (3.25)	0.220
PTA(%)	24.17 ± 8.29	29.42 ± 6.74	21.14 ± 7.56	<0.001
TBil (μmol/L)	360.65 (177.30)	310.60 (118.00)	398.0 (206.9)	<0.001
ALT(IU/L)	160.50 (443.50)	134.00 (387.50)	174.00 (509.5)	0.061
ALB(g/L)	31.50 ± 5.33	32.08 ± 5.95	31.16 ± 4.93	0.129
Amon(μmol/L)	75.00 (61.75)	64.00 (54.00)	80.00 (61.50)	0.003
BUN(μmol/L)	4.76 (4.57)	4.22 (2.63)	5.87 (6.61)	<0.001
Cr(μmol/L)	79.70 (40.75)	74.00 (23.30)	88.00 (55.50)	<0.001
GGT(IU/L)	66.50 (73.00)	68.00 (71.00)	65.00 (73.00)	0.521
AFP (ng/mL)	60.44 (172.23)	103.60 (252.39)	50.84 (124.96)	0.004
Infection (%)	83 (25.15%)	19 (15.70%)	64 (30.62%)	0.003
TNFα (ng/L)⍰	388.55 (283.34)	312.39 ± 186.68	417.94 ± 172.47	<0.001
IL10 (pg/mL)	33.23 ± 7.73	29.29 ± 6.73	35.51 ± 7.36	<0.001
iMELD socre	48.54 ± 8.62	42.51 ± 6.59	52.03 ± 7.69	<0.001
TG (mmol/L)	0.91 ± 0.15	0.92 ± 0.13	0.74 ± 0.18	0. 051
HDL-C(mmol/L)	0.29 (0.24)	0.30 (0.31)	0.26 (0.21)	0.045
LDL-C (mmol/L)	1.01 ± 0.19	1.03 ± 0.23	0.78 ± 0.17	0.069
AVT before admission(%)	10 (3.03%)	6 (4.95%)	4 (1.91%)	0.180
AVT after admission(%)	277 (83.94)	103 (85.12)	174 (83.25)	0.656
Plasmapheresis after admission(%)	151 (45.76)	54 (44.63)	97 (46.41)	0.754
Serum ApoA-V	398.58 ± 253.32	641.91 ± 168.20	257.71 ± 175.48	<0.001

**Table 2:** Multivariate analysis of laboratory variables to predict the survival of HBV-ACLF.

Variables	B	S.E.	Wald	OR (95% CI)	*P*-Value
ApoA-V	−0.006	0.001	112.033	0.994 (0.009–0.995)	0.000
iMELD socre	0.019	0.010	3.695	1.019 (1.000–1.038)	0.055
PTA	−0.047	0.010	21.449	0.954 (0.935–0.973)	0.000
AFP	−0.001	0.000	3.309	0.999 (0.998–1.000)	0.069
IL10	0.000	0.000	1.038	1.000 (1.000–1.000)	0.308
BUN	0.042	0.011	13.976	1.043 (1.020–1.066)	0.000
ApoA-I	0.176	0.386	0.208	1.193 (0.560–2.542)	0.648
Infection	−0.087	0.168	0.267	0.917 (0.659–1.275)	0.605
TBil	0.003	0.001	23.238	1.003 (1.002–1.004)	0.000
TNFα	0.000	0.000	0.765	1.000 (0.999–1.001)	0.382
Cr	−0.001	0.001	0.630	0.999 (0.997–1.001)	0.427
ApoB	0.300	0.302	0.985	1.350 (0.746–2.443)	0.321
Amon	−0.002	0.001	2.012	0.998 (0.995–1.001)	0.156
